# Perfectionism in Adolescence: Associations With Gender, Age, and Socioeconomic Status in a Norwegian Sample

**DOI:** 10.3389/fpubh.2021.688811

**Published:** 2021-08-25

**Authors:** Liv Sand, Tormod Bøe, Roz Shafran, Kjell Morten Stormark, Mari Hysing

**Affiliations:** ^1^Division of Psychiatry, Stavanger University Hospital, Stavanger, Norway; ^2^Department of Research, Helse Vest Regional Health Authority, The Western Norway Regional Health Authority, Stavanger, Norway; ^3^Department of Psychosocial Science, Faculty of Psychology, University of Bergen, Bergen, Norway; ^4^Regional Centre for Child and Youth Mental Health and Child Welfare, NORCE Norwegian Research Centre, Bergen, Norway; ^5^University College London Great Ormond Street, Faculty of Population Health Sciences, Institute of Child Health, London, United Kingdom; ^6^Department of Health Promotion and Development, Faculty of Psychology, University of Bergen, Bergen, Norway

**Keywords:** perfectionism, gender, demographic factors, Public Health, adolescence

## Abstract

**Background:** Perfectionism in adolescence has received increased attention, but few studies have examined this in non-clinical samples. This study investigated perfectionism among adolescents from the general population in relation to demographic factors.

**Methods:** The present study is cross-sectional and draws on the epidemiological youth@hordaland study. The sample consisted of 10.217 adolescents aged 16–19 years (52.9% girls). Self-reported perfectionism was assessed by the EDI-P scale from the Eating Disorder Inventory with two dimensions of perfectionism, namely self-oriented (SOP) and socially prescribed (SPP) perfectionism, and a total score. Perfectionism was analyzed in relation to age, gender, and socioeconomic status (SES) by perceived economic well-being and parental education level. Chi-squared tests, *t*-tests, and regression analyses were performed.

**Results:** There were few gender differences on the mean scores on perfectionism, with similar levels on the total score of EDI-P and SOP, while girls scored slightly higher on SPP (*p* < 0.001). The latter gender difference represented a small effect size (Cohen's *d* = 0.053). Chi-square analyses with perfectionism split at the 90th percentile across gender showed that there were significantly more girls than boys among the high scorers both for EDI-P, EDI-SOP, and EDI-SPP. There were no significant differences between levels of perfectionism between the three age groups. The logistic regression analyses adjusted by age and gender showed that adolescents with a better perceived economic well-being had increased odds of high perfectionism. This was evident for overall EDI-P (OR = 1.760, 95% CI = 1.493–2.076), SOP (OR = 1.543, 95% CI = 1.292–1.843), and SPP (OR = 1.836, 95% CI = 1.559–2.163). Parental education was not significantly associated with perfectionism scores among the adolescents.

**Conclusions:** The levels of perfectionism were relatively similar between the genders in the present study, besides slightly higher SPP among girls than boys. There were also significantly more girls than boys among the high scorers on overall perfectionism, SOP, and SPP, respectively. High perfectionism was related to SES for perceived economic well-being, but not for parental education level. Implications for further research and clinical interventions were suggested.

## Introduction

Perfectionism is traditionally defined as a tendency to set high performance standards and striving for flawlessness (1). It is commonly understood as a personality disposition developed in childhood and consolidated in adolescence as part of a more general identity formation (2). Further, perfectionism is defined as a multi-dimensional phenomenon, with self-oriented (SOP) and socially prescribed (SPP) perfectionism as two established dimensions (3). While SOP describes an inner pressure for perfection, SPP describes a subjective pressure from family, friends and others in the social environment. This suggests that perfectionism can be understood as both an intra- and interpersonal phenomenon, with standards from parents and the more general cultural context assumed to play an important role in the course of development (4). The influence from parents can be explained by social modeling, parental styles, and pressure concerning achievements (5). It has also been suggested that the dimensions of perfectionism emerge through different mechanisms, more specifically through social learning processes for SOP and social expectations processes for SPP (6).

Perfectionism can potentially have both positive and negative qualities, and one often characterizes perfectionistic traits as more or less adaptive depending on the level of self-critical indicators, perceived stress, and concerns about mistakes (7). Maladaptive perfectionism can be described as a trans-diagnostic process increasing the vulnerability for psychological distress (8), and it has been associated with both internalizing and externalizing mental health problems in adolescence (9). The dimensions of self- and socially prescribed perfectionism seem to relate differently to indicators of mental health in youth, and a study on university students showed that SPP was associated with anxiety, depression, and suicide proneness, while SOP had stronger associations with adaptive qualities such as positive self-esteem, perceived self-control, and achievement motivation (10).

There has been a growing interest for perfectionism the last three decades, presumably due to the development of multidimensional assessment instruments and a cultural focus on high achievement standards (11). Perfectionistic traits seem to become more salient among adolescents, and several longitudinal studies have reported that perfectionistic traits are on the rise among youth (4, 12). This trend together with the relation between perfectionism and psychological distress highlight the need to increase knowledge on perfectionistic traits and identify possible risk groups in general populations of adolescents.

Perfectionism has also been associated with demographic factors such as gender, age, and socioeconomic status (SES) of the family. With regard to gender differences, girls in elementary school have been shown to be more prone to perfectionistic traits than boys (13). However, this gender difference has not been reported among youth in early (14) or late adolescence (4). Yet, it has been reported that significantly more girls (15.8%) than boys (6.2%) in early adolescence showed high levels of mixed perfectionism scores (15). To our knowledge, gender differences between SOP and SPP have been studied in children (16), but not targeted specifically in adolescent samples. Further, perfectionism expressed as maladaptive evaluative concerns, but not personal standards, has been reported to be significantly higher among females than males in a sample of young adults (17).

Adolescence has been described as a sensitive period for changes in perfectionism due to a growing self-consciousness, and awareness of achievement expectations and sociocultural standards (18). Yet, earlier research on the effect of age on perfectionism has shown mixed results. Some studies have concluded that age was positively associated with both perfectionism in general and with specific dimensions of perfectionism, such as doubt about actions (19). Other studies have reported lack of significant associations between age and overall perfectionism scores (20), while expressions of perfectionism in more specific life domains has been found to be positively related to age (21). The above-mentioned studies targeted university students, and few studies have investigated the relation between perfectionism and age in adolescent populations to our knowledge (16).

SES has been positively associated with self-reported perfectionism in adolescence. More specifically, it has been suggested that affluent youth are more prone to achievement pressure, possibly due to different contributing factors with regard to the goal- and value-orientation among adolescents (22). In line with this, Lyman and Luthar (23) suggested the notion of “cost of privilege” by describing vulnerabilities with regard to psychological adjustment among academically gifted adolescents. Although they found no differences between the high and low SES group with regard to perfectionistic self-presentations, perfectionism together with peer envy and extrinsic goals explained a greater variance in internalizing and externalizing problems among affluent girls. However, this sample included youth from two extremes with regard to SES, and the findings should be interpreted with caution. Further, the sample was divided in high and low SES based on supposed family income with students from either an exclusive private school or a school with low-income students. Thus, family income was not explicitly assessed in the study, and other indicators of SES were not included (23).

While earlier research supports a sociocultural connotation of perfectionism, a majority of the studies on perfectionism and family factors has targeted specific samples of gifted students (24) or athlete groups (25). Findings from selected groups of adolescents are not necessarily representative for the general community. In addition, although youth from affluent families seem to be more vulnerable for achievement pressure and perfectionistic strivings (26), high SES has also been associated with positive outcomes in adolescence. For instance, adolescents from high SES families based on reported parental income have shown higher life satisfaction (27) and better mental health (28) than those from low SES families. Furthermore, a recent study of Norwegian youth aged 13–14 years found that maladaptive perfectionism patterns were more common among students in public schools (38%) than in private elite schools (22%) for sports and performing arts (15). The results may seem counter-intuitive given the highly competitive context of the students in elite schools. However, it is suggested that these students could be positively selected based on both their achievements as well as realistic ambitions and internalized standards driving performance motivation rather than self-doubt and perceived external pressure (15).

Thus, earlier studies on perfectionism and demographic factors have shown mixed results, both with regard to age, gender, and SES indicators. The research on perfectionism and SES also seem to have focused mainly on family income, and have not included other important SES indicators such as parental education level. Further, there has been a tendency of studying perfectionism in specific samples of gifted or affluent youth in highly competitive environments or with SES status at the extreme ends that are not necessarily representative for the general population. In addition, the dimensions of perfectionism such as SOP and SPP have been studied more extensively with regard to mental health indicators than in relation to age, gender, and SES. More knowledge on how dimensions of perfectionism present in non-clinical samples in relation to demographic variables could help identify risk groups for perfectionistic traits and support preventive and clinical interventions targeting girls and boys in adolescence.

The aim of the present study was to assess perfectionism among adolescents from a broad epidemiological study in relation to demographics defined as gender, age, and SES. Perfectionism was assessed on an overall score on EDI-P as well as on two dimensions, namely self-oriented (SOP) and socially prescribed (SPP) perfectionism. We investigated gender differences in levels of overall perfectionism, SOP, and SPP, respectively, and analyzed how age was associated with perfectionism in the total sample. Both perceived economic well-being and parental education defined indicators of SES in the study. The parents' education level is often used as an indicator of SES in surveys with adolescent samples (29), and in the present study this was measured separately for fathers and mothers. Further, the perceived economic well-being of the family has been shown to be an important variable in the pathway from SES inequalities to differences in mental health outcomes among youth (28). Based on the more consistent trends from earlier research on perfectionism and demographics, we hypothesized that girls would show generally higher scores on perfectionism than boys, and that perfectionistic traits would increase by age in the sample. Further, we expected that adolescents from high SES families would be more prone to perfectionism than adolescents from low SES families based on perceived economic well-being and/or parental education level.

## Methods

### Participants and Procedure

The sample draws on the youth@hordaland study, a broad epidemiological study assessing mental and physical health among adolescents. The sample included adolescents aged 16–19 years, *N* = 10.217, and the data were collected during spring 2012. Adolescents born between 1993 and 1995 in Hordaland county in Norway were invited with an aim to reach all adolescents in late adolescence and high school age. Adolescents in upper secondary education received study information via e-mail, and one classroom school hour was allocated for completing the questionnaire. Those not in school received information by postal mail to their home address. In addition, mental health services and other institutions (e.g., child welfare service institutions and inpatient psychiatric hospitals) were contacted to let adolescents from these settings participate. All participation was based on electronic consent from the adolescents in line with national Norwegian health authorities, stating that individuals aged 16 years or more can make decisions regarding their own health. As parents or guardians also have the right to be informed, they received written information in advance of the study.

### Self-Report Measures

Demographic variables were assessed by gender, age, ethnicity, and socioeconomic status (SES). The exact age of the participants was defined by calculating the time interval between date of birth and date of study participation. Gender was defined as either girl or boy. SES was defined as perceived economic well-being and parental education level. Perceived economic well-being was categorized in three categories as “*poorer than others*,” “*equal to others*,” or “*better than others*.” Parental education was rated by the adolescent levels separately for their mothers and fathers with the following response options: “*primary school*,” “*secondary school*,” “*college or university*<*4 years*,” “*college or university more than 4 years,”* and “*don't know*.” The responses including college or university level were combined to one category regardless of the length of education, and parental education level was then categorized as basic, intermediate, and higher, respectively.

Perfectionism was assessed by the 6-item perfectionism scale in the Eating Disorders Inventory (EDI) for children and adolescents (30). Following the multidimensional model of trait perfectionism defined by Hewitt and Flett (31), EDI-P can be divided in two dimensions, self-oriented (SOP) and socially prescribed perfectionism (SPP) (3). This two-factor model has been supported by confirmatory factor analyses in non-clinical samples of adolescents (32) as well as clinical samples with eating disorders (33).

Both subscales have three items, and they have a strong face validity as SOP refers to internal pressure *(“I hate being less than best at things,” “I feel that I must do things perfectly or not do them at all,” “I have extremely high goals”*), while SPP refers to external pressure from parents or family *(“As a child, I tried hard to avoid disappointing my parents,” “My parents have expected excellence from me,” “Only outstanding performance is good enough for my family”*).

The EDI-P items are originally rated on a 6-point Likert scale and then converted to scores from 0 to 3. In this study, the items were rated as 0 *(“not true”*), 1 *(“sometimes true”)*, or 2 *(“true”)* to adjust the categories to the broad questionnaire of youth@hordaland, allowing a range of 0–12 for the total score on EDI-P. Both EDI in general (34) and EDI-P specifically (33) have been shown to have satisfactory psychometric properties in comparable populations. The EDI-P showed an acceptable internal consistency (α = 0.74) in the sample, while it was questionable for SOP (α = 0.60) and poor for SPP (α = 0.50), respectively.

### Statistical Procedure

All statistical analyses were performed using IBM SPSS for Windows, version 24. Categorical variables were presented as percentages, while continuous variables were presented as means with standard deviations (SD). Reliability analyses of the perfectionism scale were performed by Cronbach's alpha. Descriptive statistics were performed by Pearson's Chi-squared test, independent samples *t*-tests and one-way analyses of variance (ANOVA). We calculated the effect size on the gender differences for perfectionism scores using the Cohen's *d* formula (35), with a differentiation between d's about 0.2 indicating a small effect size, about 0.5 indicating a medium effect size, and about 0.8 and higher indicating a large effect size.

Perfectionism was both used as a continuous variable in the descriptive statistics in order to compare the levels of EDI-P, EDI-SOP, and EDI-SPP across gender and age, respectively, in order to include as much variation as possible in the analyses. However, we also wanted to identify possible risk factors associated with elevated perfectionism and therefore compared low vs. high scores at the 90th percentil for the total score of EDI-P (cut-off = 8), SOP (cut-off = 4), and SPP (cut-off = 4) with regard to demographic factors. The dichotomization at the 90th percentile is well-established as a cut-off for children and adolescents with a risk of mental health problems (36). This was used in the present sample since the scoring of EDI-P was adjusted to the format of the youth@hordaland study, and the original cut-off values for the perfectionism scores could therefore not be used.

For the Chi-squared tests, we performed crosstab analyses for possible gender differences in low vs. high scores on perfectionism with *post hoc* tests using adjusted standardized residuals. In the ANOVA analyses of differences in perfectionism scores across the three age groups, we used Bonferroni *post hoc* tests and entered both linear and quadratic weighted terms to identify possible linear and/or non-linear associations. We also performed logistic regression analyses to identify the associations between SES and high perfectionism adjusted by age and gender. SES indicators were entered stepwise as categorical variables using simple effect coding with three dimensions, the middle value (“*equal”* as others for perceived economic well-being and “*intermediate”* for parental education level) defined the reference from which the other two dimensions were compared. Model 1 included perceived economic well-being, while Model 2 also comprised paternal and maternal education level, respectively. We excluded subjects with parental education level reported as “*unknown*” in the regression analyses. Due to the low proportion of incomplete responses (2.8% for EDI-P, 3.0% for perceived economic well-being, 1.8% for maternal education level, and 2.0% for paternal education level), we handled missing values by list wise deletion in the regression analyses.

## Results

### Sample Characteristics

The sample included all subjects participating in the youth@hordaland and consisted of 10.217 adolescents (53.11% girls) aged 16–19 years. Mean age was 17.43 (*SD* = 0.84). The sample formed an ethnically homogenous sample with 95.1% of the subjects born in Norway. The subjects also showed a trend toward high socioeconomic status (SES) based on the assessment of perceived economic well-being and parental education level. With regard to perceived economic well-being, 67.3% and 25.6% reported an equal or better economy than others, respectively. Concerning the parents' education level, 7.8% of the mothers were reported to have a basic education, 31.3% an intermediate education, 36.7% a higher education, and 24.2% an unknown education level. Among the fathers, the corresponding numbers were 8.0, 34.6, 31.8, and 25.7%, respectively.

### Perfectionism and Demographic Factors

We analyzed gender differences in perfectionism scores by independent samples *t*-tests comparing both EDI-P as a total score and the two dimensions comprising self-oriented perfectionism, EDI-SOP, and socially prescribed perfectionism, EDI-SPP. The analyses identified only significant differences on the EDI-SPP scores (*t* = 2.661, *p* = 0.008), with girls showing slightly higher levels than boys (see Table 1). The latter gender difference regarding SPP showed a small effect size (Cohen's *d* = 0.053).

**Table 1:** Perfectionism scores split by Gender presented as Means (SD)^n^.

**Perfectionism**	**Girls**	**Boys**
	**(*n* = 5426)**	**(*n* = 4791)**
EDI-P	4.62 (2.78)	4.56 (2.72)
EDI-SOP	2.14 (1.57)	2.16 (1.53)
EDI-SPP	2.48 (1.50)^**^	2.40 (1.50)

n *Gender differences based on independent samples t-tests*.

** *p < 0.01 for total scores on overall perfectionism (EDI-P), self-oriented perfectionism (EDI-SOP), and socially prescribed perfectionism (EDI-SPP)*.

The distribution on the single items of EDI-P was analyzed, also revealing similar response patterns for girls and boys. See Supplementary File 1 for detailed distribution of the EDI-P items across gender and for the total sample. Both genders showed the highest scores on two items (“*As a child, I tried very hard to avoid disappointing my parents*,” and “*I have extremely high goals*”). In the total sample, 27.1 and 30.5% rated these statements as true, respectively.

The perfectionism scores were also split by the 90th percentile, and Chi-square analyses across gender showed that there were significantly more girls than boys among the high scorers both for EDI-P (10.2 vs. 8.5%, *F* = 8.884, *df* = 1, *p* < 0.01), EDI-SOP (9.2 vs. 7.4%, *F* = 10.934, *df* = 1, *p* < 0.01), and EDI-SPP (10.6 vs. 9.0%, *F* = 7.236, *df* = 1, *p* < 0.01).

Further, we analyzed the association between perfectionism by EDI-P and age using one-way analyses of variance (ANOVA). There were no significant associations between levels of perfectionism and age in the sample (*F* = 1,612, *df* = 3, *p* > 0.05).

### Perfectionism and SES

We performed logistic regression analyses in order to investigate the associations between SES indicators and high scores on the perfectionism dimensions adjusted by age and gender. Perceived economic well-being was significantly associated with high perfectionism, more specifically when comparing better with equal economic well-being as others. This was evident for overall EDI-P (OR = 1.760, 95% CI = 1.493–2.076), SOP (OR = 1.543, 95% CI = 1.292–1.843), and SPP (OR = 1.836, 95% CI = 1.559–2.163). However, parental education level was not significantly associated with high perfectionism in the sample. See Supplementary File 2 for more detailed results of the regression analyses.

## Discussion

### Main Findings

The present study investigated dimensions of perfectionism in relation to demographic factors in a self-report survey among adolescents from the general population. The study design allowed an investigation of dimensions of perfectionism across gender, age, and indicators of SES including both perceived economic well-being and parental education level. We hypothesized that girls would show generally higher scores on perfectionism than boys, and that perfectionistic traits would increase by age in the sample. Further, we expected that adolescents from high SES families would be more prone to perfectionism than adolescents from low SES families. The analyses showed few gender differences regarding mean levels of perfectionism, although girls scored slightly higher on SPP than boys. Girls were also more prone to belong to the high scorers above the 90th percentile on overall perfectionism, SOP, and SPP, respectively. There were no significant differences in levels of perfectionism between the three age groups in the sample. Finally, the regression analyses revealed that perceived economic well-being, but not parental education level, was positively related to high perfectionism for both genders.

Contrary to the first hypothesis, we found few gender differences with regard to the mean levels of overall perfectionism and SOP, while girls scored slightly higher than boys on SPP. Earlier studies have shown mixed results regarding gender differences in perfectionism. It seems that the findings vary with age, with more salient gender differences among children (13) than adolescents (14). Accordingly, the meta-analysis by Curran and Hill (4) did not find evidence for a gender effect on levels of perfectionism among college students over time, for either SOP or SPP. Thus, the similarities between the genders with regard to perfectionism in the present sample are comparable to previous studies of adolescents. Yet, this might contrast to a cultural understanding of girls being more prone to perfectionistic traits than boys, possibly due to reports on gender differences from more specific samples such as music students (37). It could also be that the gender differences are more salient when comparing the high scorers on perfectionism, in line with the present findings that significantly more girls than boys were distributed above the 90th percentile for both EDI-P, EDI-SOP, and EDI-SPP, respectively. A comparable finding was reported by another Norwegian study with younger adolescents, where more girls than boys showed high levels of mixed perfectionism (15).

The mean levels of perfectionism *per se* across gender are also difficult to compare with other adolescent samples from the general population since the results are based on a different scoring than the original for EDI-2. Yet, they resemble the scores reported in another Norwegian study of high school students when converting our results to their coding of the EDI-P items (38). More specifically, with scores ranging from 1 to 3 for each item, they reported a total mean score of 11.0 (*SD* = 2.3) among girls and 13.3 (*SD* = 2.3) among boys, while the corresponding levels in the present sample were comparable to these with converted mean scores of 10.62 (*SD* = 2.79) among girls and 10.56 (*SD* = 2.73) among boys. Interestingly, the study by Ytterdal (38) found a significant gender effect with higher scores on overall perfectionism among boys than girls, which was not the case in the present study.

In contrast to our second hypothesis, we found no significant differences between levels of perfectionism in the three age groups in the sample. Earlier studies on the association between perfectionism and age have also shown mixed results. It has been reported that age showed a positive association with both overall perfectionism and specific dimensions of perfectionism (19) as well as with more specific life domains (21). Further, it has been shown that perfectionism seem to decrease after adolescence (39). Thus, the lack of significant age effects in the present sample could be due to the overall investigation of perfectionism instead of domain-specific measures (40), and the possibility that perfectionistic traits are more stable in late adolescence than in the transition to adulthood. It could also be that developmental trajectories of perfectionism reveal more pronounced age differences than dimensions of perfectionism measured at one point of time (41).

In support of our third hypothesis, we found that high scores on perfectionism were positively associated with perceived economic well-being, but not with parental education level. Accordingly, previous studies have reported of elevated perfectionistic tendencies among affluent girls (23), and it has been suggested that this could be explained by more pronounced achievement- and goal-orientation in high income families (22). However, this does not necessarily imply that elevated perfectionism among adolescents from high SES groups is maladaptive, as this probably depends on both the level and quality of the perfectionistic traits. In accordance to this, when specifying perfectionism in healthy and unhealthy patterns in a Norwegian study, adolescents from public schools showed higher levels of maladaptive patterns than students in private elite schools from supposedly high income families (15). Since the current study did not categorize adaptive or maladaptive qualities of perfectionism, the results should be interpreted with caution with regard to a potential burden or a “cost of privilege” (23) among the high scorers of EDI-P, SOP, and SPP. Yet, the present findings add important knowledge to the relation between perfectionistic and demographic variables in adolescence and highlight SES as a more significant variable than gender and age.

### Study Strengths and Limitations

Perfectionism has received increased attention in the field of mental health among adolescents, but few studies have investigated how this presents in general samples. The strength of the present study was to investigate perfectionism in a broad and representative sample of Norwegian adolescents by self-reported data in relation to demographic variables, reinforcing external validity and generalizability. The online questionnaire was administered individually, possibly reducing potential response biases in the sample. However, although adolescents could complete the questionnaire at any time during the data collection period, it is likely that adolescents who frequently attend school were present during the allocated time for data collection and were more likely to participate.

Further, we analyzed perfectionism by EDI-P with an overall score as well as sub-scales defined as SOP and SPP, and this reflects the established understanding of perfectionism as a multi-dimensional phenomenon. Yet, the scoring of the perfectionism scale were adjusted to the format of the youth@hordaland study, and the results were therefore more difficult to compare with other studies with adolescent samples. Further, the perfectionism scale did not classify the perfectionistic traits as adaptive or maladaptive patterns nor connect the scores to specific life domains. Thus, with such an overall measure of perfectionism, we might underreport associations with gender and age that other studies using more detailed perfectionism scales have presented. Additionally, the internal consistency of the SOP and SPP was questionable in the present study. However, it has been reported higher reliability on the EDI-2 in non-clinical and adult samples compared to population-based and adolescent samples (42). This could explain the relatively low Cronbach's alpha for the perfectionism subscales in this sample. It is recommended to assess and report the internal consistency when using EDI-2, as the reliability seem to vary according to sample characteristics (42).

Furthermore, the association between perfectionism and SES seems to be influenced by variables that the present study did not include, such as achievement- and goal-orientation (22), or parental perfectionism (43). The data were also collected in 2012, perhaps reflecting self-reported perfectionism among adolescents with less exposure to achievement pressure than more recent studies on youth. Yet, the focus on perfectionism and social standards was already relevant for the cultural context at that point of time (11), supporting the representability of the present findings.

It could also be noted that the quality of adolescents' rating of the education level of their parents is questionable and would perhaps be more consistent if assessed across measurement occasions (29). In line with this, the measure on perceived economic well-being was a subjective rating rather than an objective measure of the family's economic circumstances, but the relative ratings corresponds fairly well to ranks of household income (44). Finally, using a cross-sectional design does not allow causal interpretations of the association between the targeted variables.

### Implications for Research and Clinical Interventions

The research on perfectionism in adolescence has followed advances in multi-dimensional measures and a cultural focus on high achievements standards that have caused concerns with regard to perceived performance pressure and mental health problems among youth. While previous studies of perfectionism have mainly targeted specific risk groups in high-achieving environments, recent studies have included broader samples and/or compared risk and control groups among adolescents. It becomes evident that specific risk groups and general samples show both similarities and differences with regard to how perfectionism expresses and relates to a potential psychological burden. Accordingly, future research on perfectionism could further explore the shared and specific vulnerability in defined risk groups and general adolescent samples with regard to both demographic factors, perceived performance pressure, and mental health indicators. Finally, longitudinal studies should be planned in order to gain insight on possible developmental pathway for perfectionism from early to late adolescence together with both individual, familiar, and culturally defined indicators. In line with this, one could also elaborate the theoretical framework for perfectionistic traits and differentiate mechanisms that may be relevant for dimensions of perfectionism, where social learning and expectations processes has been suggested for SOP and SPP, respectively (6).

Perfectionism seems to have increased internationally through the last decades among adolescents (4), suggesting that youth today are more demanding of themselves as well as believing that others are more demanding of them. In light of these trends, maladaptive perfectionism should be targeted in selected programs for youth in order to prevent the development of psychological distress and interpersonal problems later in age. Based on the present findings, both girls and boys in adolescence should be included, with a special attention on affluent youth and girls with elevated perfectionism scores. There are several programs available, but only some have evaluated the effect systematically. One targeted intervention for perfectionism showed positive effect of a short intervention aimed at skills to reduce perfectionistic traits in community sample of adolescents (45). There are also modules for treating clinical perfectionism within a cognitive behavioral framework, both with regular therapist support or by internet-based interventions (46), or in combination with a diagnosed eating disorder (47).

Additionally, an increased knowledge on adaptive qualities of perfectionism is important in order to support a positive development among adolescents. The distinction between perfectionistic *concerns* and *strivings* could be useful in this context (48), concluding that perfectionistic concerns over mistakes and negative evaluations from others are associated with higher risk of mental health problems, while perfectionistic strivings are associated with psychological adjustment, positive affect, and life satisfaction. Thus, adaptive perfectionism should be supported in adolescence in order to build endurance, active coping styles, and intrinsic motivation to achieve within realistic standards.

## Conclusions

The present study targeted self-reported perfectionism in relation to demographic factors among adolescents in a broad epidemiological sample. SES indicators included both perceived economic well-being and parental education level. The mean scores on perfectionism did not differ considerably by gender or age. Yet, girls were more prone to belong to the high scorers on both overall perfectionism and the dimensions of SOP and SPP than boys, respectively. High perfectionism scores were also positively associated with perceived economic well-being, while the association with parental education did not prove significant. The findings add important nuances to the relation between perfectionism and demographic variables in adolescence. Implications for further research and preventive interventions were suggested, with a focus on gaining more insight to developmental pathways for perfectionism through longitudinal studies, targeting maladaptive perfectionistic traits in preventive and clinical interventions, and supporting adaptive qualities of perfectionism in adolescence.

## Data Availability Statement

The datasets presented in this article are not readily available because of the privacy restrictions in accordance with the ethical approval for the youth@hordaland-survey. Requests to access the datasets should be directed to the Bergen Child Study group.

## Ethics Statement

The studies involving human participants were reviewed and approved by Regional Committee for Medical and Health Research Ethics in Western Norway. Written informed consent to participate in this study was provided by the participants' legal guardian/next of kin. All participation was based on electronic consent from the adolescents in line with national Norwegian health authorities, stating that individuals aged 16 years or more can make decisions regarding their own health. As parents or guardians also have the right to be informed, they received written information in advance of the study.

## Author Contributions

MH, KS, and TB were involved in the data acquisition for youth@hordaland, while LS and MH were responsible for planning the design of the present study. LS conducted the statistical analyses and conferred with MH and TB regarding the results. LS drafted the manuscript, while RS and KS contributed to specifying the research questions as well as interpreting the results. All authors have revised the manuscript critically, and they have read and approved the final manuscript.

## Author Disclaimer

The views expressed are those of the authors and not necessarily those of the NHS, the NIHR or the Department of Health.

## Conflict of Interest

The authors declare that the research was conducted in the absence of any commercial or financial relationships that could be construed as a potential conflict of interest.

## Publisher's Note

All claims expressed in this article are solely those of the authors and do not necessarily represent those of their affiliated organizations, or those of the publisher, the editors and the reviewers. Any product that may be evaluated in this article, or claim that may be made by its manufacturer, is not guaranteed or endorsed by the publisher.

## Abbreviations

BCS: Bergen Child Study
EDI-P: Eating Disorders Inventory Perfectionism subscale
SES: Socioeconomic Status
SOP: Self-Oriented Perfectionism
SPP: Socially Oriented Perfectionism.

## References

[1] Hewitt PL Norton GR Flett GL Callander L Cowan T. Dimensions of perfectionism, hopelessness, and attempted suicide in a sample of alcoholics. Suicide Life Threat Behav. (1998) 28:395–406. 9894307

[2] Negru-Subtirica O Pop EI Damian LE Stoeber J. The very best of me: longitudinal associations of perfectionism and identity processes in adolescence. Child Dev. (2021). 10.1111/cdev.13622 34231882

[3] Sherry SB Hewitt PL Besser A McGee BJ Flett GL. Self-oriented and socially prescribed perfectionism in the eating disorder inventory perfectionism subscale. Int J Eat Disord. (2004) 35:69–79. 10.1002/eat.10237 14705159

[4] Curran T Hill AP. Perfectionism is increasing over time: a meta-analysis of birth cohort differences from 1989 to 2016. Psychol Bull. (2017) 145, 410–29. 10.1037/bul0000138 29283599

[5] Stoeber J Childs JH. Perfectionism. In: Levesque RJR, editor. Encyclopedia of Adolescence. New York, NY: Springer (2011). p. 2053–9. 10.1007/978-1-4419-1695-2_279

[6] Damian LE Stoeber J Negru O Baban A. On the development of perfectionism in adolescence: perceived parental expectations predict longitudinal increases in socially prescribed perfectionism. Pers Individ Dif. (2013) 55:688–93. 10.1016/j.paid.2013.05.021

[7] Rice KG Richardson CM. Classification challenges in perfectionism. J Couns Psychol. (2014) 61:641–8. 10.1037/cou0000040 25111705

[8] Egan SJ Wade TD Shafran R. Perfectionism as a transdiagnostic process: a clinical review. Clin Psychol Rev. (2011) 31:203–12. 10.1016/j.cpr.2010.04.009 20488598

[9] Taylor EP Couper R Butler CM. Adolescent perfectionism: structural features of the Frost Multidimensional Perfectionism Scale and correlates with attachment and psychopathology. Psychol Psychother. (2017) 90:686–704. 10.1111/papt.12133 28585772

[10] Klibert JJ Langhinrichsen-Rohling J Saito M. Adaptive and maladaptive aspects of self-oriented versus socially prescribed perfectionism. J Coll Stud Dev. (2005) 46:141–56. 10.1353/csd.2005.0017

[11] Flett GL Hewitt PL. Reflections on three decades of research on multidimensional perfectionism: an introduction to the special issue on further advances in the assessment of perfectionism. J Psychoeducational Assess. (2020) 38:3–14. 10.1177/0734282919881928

[12] Portesova S Urbanek T. Typology of perfectionism in a group of mathematically gifted Czech adolescents over one decade. J Early Adolesc. (2013) 33:1116–44. 10.1177/0272431613487603

[13] Bas AU. Dimensions of perfectionism in elementary school-aged children: associations with anxiety, life satisfaction, academic achievement. Egitim Ve Bilim-Educ Sci. (2011) 36:261–72.

[14] Rice KG Leever BA Noggle CA Lapsley DK. Perfectionism and depressive symptoms in early adolescence. Psychol Sch. (2007) 44:139–56. 10.1002/pits.20212

[15] Stornaes AV Rosenvinge JH Sundgot-Borgen J Pettersen G Friborg O. Profiles of perfectionism among adolescents attending specialized elite- and ordinary lower secondary schools: a Norwegian cross-sectional comparative study. Front Psychol. (2019) 10:2039. 10.3389/fpsyg.2019.02039 31551878 PMC6743349

[16] Melero S Morales A Espada JP Fernandez-Martinez I Orgiles M. How does perfectionism influence the development of psychological strengths and difficulties in children? Int J Environ Res Public Health. (2020) 17:4081. 10.3390/ijerph17114081 32521665 PMC7312165

[17] Riviere J Douilliez C. Perfectionism, rumination, and gender are related to symptoms of eating disorders: a moderated mediation model. Pers Individ Dif. (2017) 116:63–8. 10.1016/j.paid.2017.04.041

[18] Flett GL Hewitt PL Oliver JM MacDonald S. Perfectionism in children and their parents: a developmental analysis. In: Hewitt GLF, editor. Perfectionism: Theory, Research, Treatment. Washington, DC: American Psychological Association (2002). p. 89–132. 10.1037/10458-004

[19] Butt FM. The role of perfectionism in psychological health: a study of adolescents in Pakistan. Eur J Psychol. (2010) 6:125–47. 10.5964/ejop.v6i4.227

[20] Schweitzer RD Hamilton TK. Perfectionism and mental health in Australian university students: is there a relationship? J Coll Stud Dev. (2002) 43:684–95.

[21] Stoeber J Stoeber FS. Domains of perfectionism: prevalence and relationships with perfectionism, gender, age, and satisfaction with life. Pers Individ Dif. (2009) 46:530–5. 10.1016/j.paid.2008.12.006

[22] Travers LV Bohnert AM Randall ET. Brief report: adolescent adjustment in affluent communities: the role of motivational climate and goal orientation. J Adolesc. (2013) 36:423–8. 10.1016/j.adolescence.2012.11.009 23351983

[23] Lyman EL Luthar SS. Further evidence on the “costs of privilege”: perfectionism in high-achieving youth at socioeconomic extremes. Psychol Sch. (2014) 51:913–30. 10.1002/pits.21791 26345229 PMC4559285

[24] Mofield EL Peters MP. Multidimensional perfectionism within gifted suburban adolescents: an exploration of typology and comparison of samples. Roeper Rev J Gifted Educ. (2015) 37:97–109. 10.1080/02783193.2015.1008663

[25] Ommundsen Y Roberts GC Lemyre PN Miller BW. Peer relationships in adolescent competitive soccer: associations to perceived motivational climate, achievement goals and perfectionism. J Sports Sci. (2005) 23:977–89. 10.1080/02640410500127975 16195049

[26] Luthar SS Becker BE. Privileged but pressured? a study of affluent youth. Child Dev. (2002) 73:1593–610. 10.1111/1467-8624.00492 12361321 PMC3524830

[27] Ozdemir Y. Examining the subjective well-being of adolescents in terms of demographic variables, parental control, parental warmth. Egitim Ve Bilim-Edu Sci. (2012) 37:20–33.

[28] Bøe T Dearing E Stormark KM Zachrisson HD. Subjective economic status in adolescence: determinants and associations with mental health in the Norwegian Youth@Hordaland Study. J Fam Econ Issues. (2018) 39:323–36. 10.1007/s10834-017-9553-4

[29] Aaro LE Flisher AJ Kaaya S Onya H Namisi FS Wubs A. Parental education as an indicator of socioeconomic status: improving quality of data by requiring consistency across measurement occasions. Scand J Public Health. (2009) 37:16–27. 10.1177/1403494808086917 19493978

[30] Garner DM. The Eating Disorder Inventory-2. Odessa: FL: Psychological Assessment Resources (1991).

[31] Hewitt PL Flett GL. Perfectionism in the self and social contexts - conceptualization, assessment, and association with Psychopathology. J Pers Soc Psychol. (1991) 60:456–70. 10.1037/0022-3514.60.3.456 2027080

[32] Muro-Sans P Amador-Campos JA Pero-Cebollero M. Factor structure of eating disorders inventory-2 in a Spanish sample. Eat Weight Disord. (2006) 11:e42–52. 10.1007/BF03327759 16809969

[33] Lampard AM Byrne SM McLean N Fursland A. The eating disorder inventory-2 perfectionism scale: factor structure and associations with dietary restraint and weight and shape concern in eating disorders. Eat Behav. (2012) 13:49–53. 10.1016/j.eatbeh.2011.09.007 22177396

[34] Engelsen BK Laberg JC. A comparison of three questionnaires (EAT-12, EDI. and EDE-Q) for assessment of eating problems in healthy female adolescents. Nord J Psychiatry. (2001) 55:129–35. 10.1080/08039480151108589 11802911

[35] Cohen J. Statistical Power Analysis for the Behavioral Sciences (2nd Edn.). Hillsdale, NJ: Erlbaum (1988).

[36] Goodman R. Psychometric properties of the strengths and difficulties questionnaire. J Am Acad Child Adolesc Psychiatry. (2001) 40:1337–45. 10.1097/00004583-200111000-00015 11699809

[37] Patston T Osborne M. The developmental features of music performance anxiety and perfectionism in school age music students. Perform Enhancement Health. (2016) 4:42–9. 10.1016/j.peh.2015.09.003

[38] Ytterdal M. The correlation between perfectionism, stress, coping and physical fitness among high school students (Master). Western Norway University of Applied Sciences, Bergen (MFAKS514) (2018).

[39] Landa CE Bybee JA. Adaptive elements of aging: self-image discrepancy, perfectionism, eating problems. Dev Psychol. (2007) 43:83–93. 10.1037/0012-1649.43.1.83 17201510

[40] Dunn JGH Dunn JC McDonald K. Domain-specific perfectionism in intercollegiate athletes: relationships with perceived competence and perceived importance in sport and school. Psychol Sport and Exerc. (2012) 13:747–55. 10.1016/j.psychsport.2012.05.002

[41] Vaillancourt T Haltigan JD. Joint trajectories of depression and perfectionism across adolescence and childhood risk factors. Dev Psychopathol. (2018) 30:461–77. 10.1017/S0954579417000979 28606188

[42] Gleaves DH Pearson CA Ambwani S Morey LC. Measuring eating disorder attitudes and behaviors: a reliability generalization study. J Eat Disord. (2014) 2:6. 10.1186/2050-2974-2-6 24764530 PMC3984738

[43] Randall ET Bohnert AM Travers LV. Understanding affluent adolescent adjustment: the interplay of parental perfectionism, perceived parental pressure, and organized activity involvement. J Adolesc. (2015) 41:56–66. 10.1016/j.adolescence.2015.03.005 25828548

[44] Bøe T Petrie KJ Sivertsen B Hysing M. Interplay of subjective and objective economic well-being on the mental health of Norwegian adolescents. SSM Popul Health. (2019) 9:100471. 10.1016/j.ssmph.2019.100471 31720359 PMC6839012

[45] Bento C Pereira AT Roque C Tavares Saraiva JM Ferreira Macedo E Santos AJ. Longitudinal effects of an intervention on perfectionism in adolescents. Psicothema. (2017) 29:317–22. 10.7334/psicothema2016.223 28693700

[46] Zetterberg M Carlbring P Andersson G Berg M Shafran R Rozental A. Internet-based cognitive behavioral therapy of perfectionism: comparing regular therapist support and support upon request. Internet Interv. (2019) 17:100237. 10.1016/j.invent.2019.02.001 30891422 PMC6403448

[47] Cooper Z Stewart H. CBT-E and the younger patient. In: Fairburn CG, editor. Cognitive Behaviour Therapy and Eating Disorders. New York, NY: The Guilford Press (2008). p. 221–30.

[48] Stoeber J Otto K. Positive conceptions of perfectionism: approaches, evidence, challenges. Pers Soc Psychol Rev. (2006) 10:295–319. 10.1207/s15327957pspr1004_2 17201590

