# Supplementary material for: Perfectionism in Adolescence: Associations With Gender, Age, and Socioeconomic Status in a Norwegian Sample
Source: Front Public Health. 2021 Aug 25;9:688811. doi: 10.3389/fpubh.2021.688811 (PMC8424040; doi:10.3389/fpubh.2021.688811)
Supplement: Supplementary file 1 [file Table_1.DOCX]

**Supplementary file 1**

The distribution of EDI-P Items across Gender and for Total Sample

|  | **Girls (n = 5426)** | | | **Boys (n = 4791)** | | | | **Total sample (10.217)** | | | |
| --- | --- | --- | --- | --- | --- | --- | --- | --- | --- | --- | --- |
| **EDI-P Items** | (1) | (2) | (3) | | (1) | (2) | (3) | | (1) | (2) | (3) |
| “As a child, I tried very hard to avoid dissappointing my parents” | 17.5 % | 52.5 % | 30.0 % | | 23.6 % | 52.4 % | 24.0 % | | 20.4 % | 52.4 % | 27.2 % |
| “I hate being less than best at things” | 45.8 % | 38.7 % | 15.5 % | | 40.3 % | 39.9 % | 19.8 % | | 43.2 % | 39.3 % | 17.5 % |
| “My parents have expected excellence from me” | 58.5 % | 29.8 % | 11.7 % | | 53.2 % | 33.8 % | 13.0 % | | 56.0 % | 31.7 % | 12.3 % |
| “I feel that I must do things perfectly or not do them at all” | 70.6 % | 21.7 % | 7.7 % | | 72.2 % | 22.2 % | 5.6 % | | 71.4 % | 21.9 % | 6.7 % |
| “I have extremely high goals” | 24.2 % | 44.1 % | 31.7 % | | 25.1 % | 45.9 % | 29.0 % | | 24.6 % | 44.9 % | 30.5 % |
| “Only outstanding performance is good enough for my family” | 37.6 % | 42.7% | 19.7 % | | 38.9 % | 42.4 % | 18.7 % | | 38.2 % | 42.6 % | 19.2 % |

*Note* ^n^*:* The scoring on EDI-P is based on the following responses: (1): Not true, (2): Partly true, and (3): True.
