# Supplementary material for: Perfectionism in Adolescence: Associations With Gender, Age, and Socioeconomic Status in a Norwegian Sample
Source: Front Public Health. 2021 Aug 25;9:688811. doi: 10.3389/fpubh.2021.688811 (PMC8424040; doi:10.3389/fpubh.2021.688811)
Supplement: Supplementary file 2 [file Table_2.DOCX]

**Supplementary file 2**

|  |  | **Model 1** | | **Model 2** | |
| --- | --- | --- | --- | --- | --- |
|  |  | OR | 95 % CI | OR | 95 % CI |
| **EDI-P** | Economic wellbeing  Poorer  Better | 1.070  1.793*** | 0.769-1.490  1.524-2.110 | 1.076  1.760*** | 0.771-1.501  1.493-2.076 |
|  | Paternal education  Basic  Higher |  |  | 1.081  1.089 | 0.821-1.423  0.905-1.309 |
|  | Maternal education  Basic  Higher |  |  | 1.096  1.116 | 0.825-1.457  0.928-1.342 |
| **EDI-SOP** | Economic wellbeing  Poorer  Better | 1.329  1.587*** | 0.964-1.832  1.332-1.891 | 1.333  1.543*** | 0.965-1.843  1.292-1.843 |
|  | Paternal education  Basic  Higher |  |  | 1.293  1.158 | 0.975-1.713  0.951-1.411 |
|  | Maternal education  Basic  Higher |  |  | 0.051  1.169 | 0.777-1.423  0.961-1.423 |
| **EDI-SPP** | Economic wellbeing  Poorer  Better | 0.933  1.818*** | 0.659-1.321  1.547-2.136 | 0.909  1.673*** | 0.641-1.290  1.559-2.163 |
|  | Paternal education  Basic  Higher |  |  | 1.096  1.023 | 0.839-1.431  0.850-1.231 |
|  | Maternal education  Basic  Higher |  |  | 1.213  0.980 | 0.927-1.588  0.816-1.178 |

SES Indicators associated with Perfectionism defined as EDI-P, EDI-SOP, and EDI-SPP^n^

*Note*^n^: Results for logistic regression analyses for high levels of perfectionism above 90^th^ percentile and SES indicators defined as perceived economic wellbeing (*equal* as reference), paternal and maternal education level (*intermediate* as reference) adjusted by age and gender with p-values at *** <.000.
